# Osteogenesis Imperfecta, Pseudoachalasia, and Gastric Cancer

**DOI:** 10.1155/2015/685459

**Published:** 2015-03-22

**Authors:** Dilsa Mizrak, Ali Alkan, Batuhan Erdogdu, Gungor Utkan

**Affiliations:** ^1^Department of Medical Oncology, Ankara University School of Medicine, 06300 Ankara, Turkey; ^2^Department of Internal Medicine, Ankara University School of Medicine, 06300 Ankara, Turkey

## Abstract

Osteogenesis imperfecta (OI) is a rare, inherited skeletal disorder characterized by abnormalities of type 1 collagen. Malignancy is rarely reported in patients with OI and it was suggested that this disease can protect against cancer. Here, we report a 41-year-old woman with symptoms of achalasia where repeated treatment of pneumatic dilation and stent replacement was unsuccessful; therefore, surgery was performed. Pathology showed gastric adenocarcinoma unexpectedly. Chemotherapy was given after assessing dihydropyrimidine dehydrogenase (DPD) enzyme activity, which can be deficient in OI patients. This is the first report of gastric cancer mimicking achalasia in a patient with OI.

## 1. Introduction

Osteogenesis imperfecta (OI) is a heterogeneous group of disorders, predominantly characterized by osteopenia with liability to fracture. The disorder is frequently associated with blue sclera, dental abnormalities, progressive hearing loss, and a positive family history [1]. Severity of the disease is very variable with one type which is lethal prenatally. The other type with mild features makes distinguishability of affected individuals harder from normal individuals. The overall prevalence of OI is 1-2 per 20,000 births [2]. Cancer is not frequently seen in OI patients, and in particular epithelial cancers are rarely reported in OI patients [3]. We report the first patient with OI, presented as pseudoachalasia and diagnosed as gastric cancer.

## 2. Case Report

**Figure 1:**
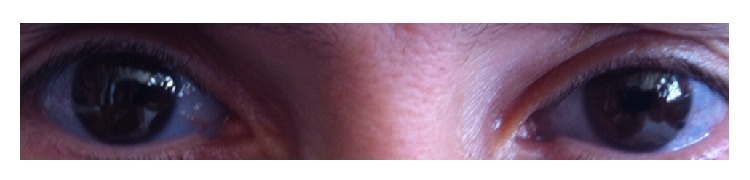
Blue sclera.

**Figure 2:**
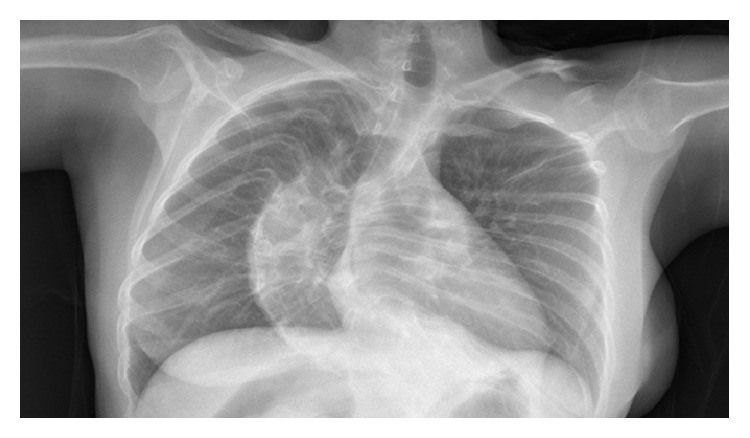
Widened mediastinum with severe scoliosis.

**Figure 3:**
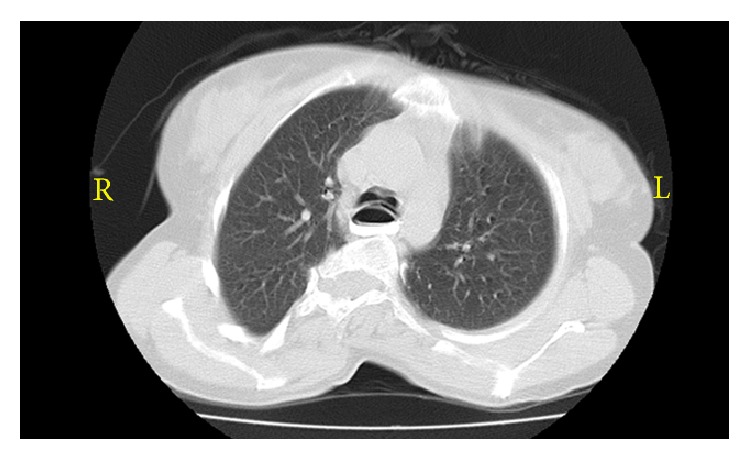
Widened esophagus.

A 41-year-old woman with a short stature and blue sclera (Figure 1) was admitted to our hospital 2 years ago, with difficulty in swallowing and weight loss. We learned that she had a family history of OI and had the diagnosis of OI when she was a child. The chest radiograph showed a widened mediastinum (Figure 2) and computer tomography showed a widened esophagus (Figure 3). There was no detectable lesion by endoscopy. Esophagogram showed a dilated esophagus with an air-fluid level, which can be seen in achalasia. Confirmation was made by esophageal manometry which showed incomplete relaxation of the lower esophageal sphincter. Repeated treatment of pneumatic dilation and stent replacement was unsuccessful. Biopsy which was made from distal esophagus was reported as esophagitis. Due to the continuation of symptoms, esophagectomy and proximal gastrectomy were performed and pathology report showed adenocarcinoma, grade 2, and tumor infiltrated to serosa (T3N1M0). Because the tumor was positive in the proximal surgical margin, second surgery was performed. The anatomy of abdomen was not suitable for radiation therapy. Since the patient was in high risk group, docetaxel + 5-fluorouracil + folinic acid treatment in the adjuvant setting for 6 cycles was given. No significant adverse effect related to the chemotherapy was seen. In the literature, DPD enzyme deficiency and related toxicity secondary to 5-FU are reported for OI patients; therefore, we studied DPD enzyme activity before administration of 5-FU and found no lack of DPD enzyme activity. The patient completed her therapy 6 months ago and is still in remission without any symptoms.

## 3. Discussion

OI is a rare disorder, in which a mutation of COL1A1 and COL1A2 genes that encode the procollagen chains of type I collagen takes place. These mutations can have profound effect on extracellular matrix and give rise to increased bone fragility and low bone mass [2]. Type of mutation determines the severity of the disease. It may be sufficient to classify patients simply as mild (type I), lethal (type II), and moderately severe (type III), ranging from intrauterine fractures to very mild forms without any fracture and hard to distinguish from a healthy person [2]. Diagnosis of the disease can be done clinically for most patients. Short stature, blue sclera, dentinogenesis imperfecta, hearing impairment, and bone fractures are the most common characteristics of the disease.

Concurrent cancer is rarely seen in OI patients and it was suggested by Rosenstock et al. that these patients have cancer protection, but we have no evidence and such mechanism is not known. Osteosarcoma is the most reported malignancy in OI patients [4, 5]. It is suggested that it can be due to cumulative radiation because these patients have numerous diagnostic radiographs of fractured bones. Metal implants used for fractured bones and repeated trauma can also be the etiologic factor for osteosarcoma in OI patients. Among epithelial tumors, reported malignancies are breast [6, 7], ovarian [8], colon [9], and gastric cancers [10].

Treatment decision should be done carefully for these patients due to the severe 5-fluorouracil (FU) toxicity reported in OI patients [7]. 5-FU is the mainstay of chemotherapy in gastric cancer; therefore, we studied DPYP∗2A before starting the therapy and found no dysfunction of the DPD enzyme. Our patient completed the therapy without any significant adverse effect.

Achalasia is the most common primary esophageal motor disorder and etiology is unknown. It is characterized by the degeneration of Auerbach's plexus; however, changes may also occur in the vagus nerve and the swallowing center. It is characterized with painless dysphagia and weight loss. Malignancy should be kept in mind if weight loss exceeds 10% of the body weight. Pseudoachalasia is a disorder that mimics clinical, radiographic, and manometric findings of achalasia as a result of another underlying disorder like adenocarcinoma of the cardia. This is the first report of concurrent OI, pseudoachalasia, and gastric cancer.

Because malignancy is rarely reported but can be seen in OI patients, one should be careful when evaluating symptoms of patients with OI. Also, it is important to evaluate symptoms mimicking achalasia, which can be seen in adenocarcinoma of cardia.
